# Proteomic approach to understand the molecular physiology of symbiotic interaction between *Piriformospora indica* and *Brassica napus*

**DOI:** 10.1038/s41598-018-23994-z

**Published:** 2018-04-10

**Authors:** Neeraj Shrivastava, Li Jiang, Pan Li, Archana Kumari Sharma, Xingyuan Luo, Sanling Wu, Rashmi Pandey, Qikang Gao, Binggan Lou

**Affiliations:** 1 Institute of Biotechnology, Zhejiang University, Hangzhou, 310058 China; 2 Institute of Insect Sciences, Zhejiang University, Hangzhou, 310058 China; 3 Centre for Environmental Science and Technology, Central University of Punjab, Bathinda, 151001 India; 4 Hangzhou No. 14 High School, Hangzhou, 310006 China; 5 Analysis Center of Agrobiology and Environmental Sciences, Zhejiang University, Hangzhou, 310058 China; 6 Government Kanya Ashram School, Aara, Jashpur, Chhattisgarh 496331 India; 7 Amity Institute of Microbial Technology, Amity University Uttar Pradesh, Noida, UP 201303 India

## Abstract

Many studies have been now focused on the promising approach of fungal endophytes to protect the plant from nutrient deficiency and environmental stresses along with better development and productivity. Quantitative and qualitative protein characteristics are regulated at genomic, transcriptomic, and posttranscriptional levels. Here, we used integrated in-depth proteome analyses to characterize the relationship between endophyte *Piriformospora indica* and *Brassica napus* plant highlighting its potential involvement in symbiosis and overall growth and development of the plant. An LC-MS/MS based label-free quantitative technique was used to evaluate the differential proteomics under *P. indica* treatment vs. control plants. In this study, 8,123 proteins were assessed, of which 46 showed significant abundance (34 downregulated and 12 upregulated) under high confidence conditions (p-value ≤ 0.05, fold change ≥2, confidence level 95%). Mapping of identified differentially expressed proteins with bioinformatics tools such as GO and KEGG pathway analysis showed significant enrichment of gene sets involves in metabolic processes, symbiotic signaling, stress/defense responses, energy production, nutrient acquisition, biosynthesis of essential metabolites. These proteins are responsible for root’s architectural modification, cell remodeling, and cellular homeostasis during the symbiotic growth phase of plant’s life. We tried to enhance our knowledge that how the biological pathways modulate during symbiosis?

## Introduction

The vegetable oil production has been crossed approximately 87 million metric tons every year. The worldwide consumption level of vegetable oils, since last decade, enhanced almost 50% with the doubled price^1,2^. The Rapeseed/canola (*Brassica napus*) occupied second place (13.6%) in global oilseed production and third place in the global vegetable oil consumption. The annual planting acreage is about 8 MH (million hectares) in China, which accounts for a quarter of the world’s total *B. napus* production^3,4^.

Increasing the high yield capacity of *B. napus* is the big challenge in current agriculture research^3^. The yield is restricted by various environmental factors like an intensification of land use, irrigation thorough metal/pesticide-contaminated water, water deficit, high temperature, drought, salinity and fungal pathogens^3,4^. Keeping in mind the environmental aspects, root endophytes and mycorrhiza seems to be a promising approach to cope up with this problem without affecting the soil environment. Subsequently, endophytes help in improving the plant health, growth, development and productivity. These endophytes receive essential organic carbon forms from the host for survival. In return, they secrete some enzymes, which help in solubilizing some complex soil nutrients to available forms for plant uptake. Thus, fungus supports enhanced water and nutrient uptake, protect from various biotic, abiotic stresses and plant pathogens for better growth of the host plant^5–8^.

*Piriformosopra indica*, a mycorrhiza like phyto-promotional root endosymbiont, was isolated from woody shrubs *Prosopisjuliflora* (Swartz) DC. and *Zizyphusnummularia* (Burm. fil.) Wt. &Arn.in Thar desert of Indian province Rajasthan^9^. An extensive research has been conducted on this fungus since last two decades. *P. indica* confers enhanced vegetative growth, stress tolerance, resistance to plant pathogens in wide host range from monocot to dicot plant species without host specificity. The fungus grows inside the root cortex of host plants without invading the endodermis and aerial parts of the plants^6–8,10,11^. Previously our group has studied the interaction between *P. indica* and *B. napus*, resulted in significant enhancement of agronomic parameters, biomass and nutrient elements contents. Quality parameters of oilseed quality like oils content and other acid contents were also found increased significantly^12^.

Previously in past few years, limited researchers worked on symbiosis at the molecular level, especially at the protein part. Song *et al*.^13^ investigated the proteomes of *Glomus mosseae* and *Amorphafruticosa* interaction. Expressed proteins found extensively involved in several biological and cellular processes^14^. Focusing on plant–microbe interactions, Vannini *et al*.^15^ analyzed the proteomic profile and investigated the complex inter-domain network during the interaction of arbuscular mycorrhizal fungus (AMF) *Gigaspora margarita* with *Lotus japonicas*^15^. Proteomes of two wheat cultivars interacted with *G. mosseae* indicated 55 and 66 DEPs in both, resulting in modulation of proteins related to metabolism^14^. In another study, leaf proteomics of *P. indica* colonized barley plant under salt and water stress stated that *P. indica* induces a systemic response by altering physiological and metabolic capacity in colonized plants improving tolerance to stresses^16,17^.

The aim of this study was to analyze the protein expression pattern during symbiotic association of fungal root endophyte and host plant. This study supports the idea that proteomics may be closer to phenotype and may incorporate the new insights into plant-microbe interaction explaining ‘how fungal partner provide direct/indirect benefits to the host plant’. Here, we applied label-free quantitative proteomics to investigate the differences in the protein expression between *P. indica* colonized and control plants.

**Figure 1:**
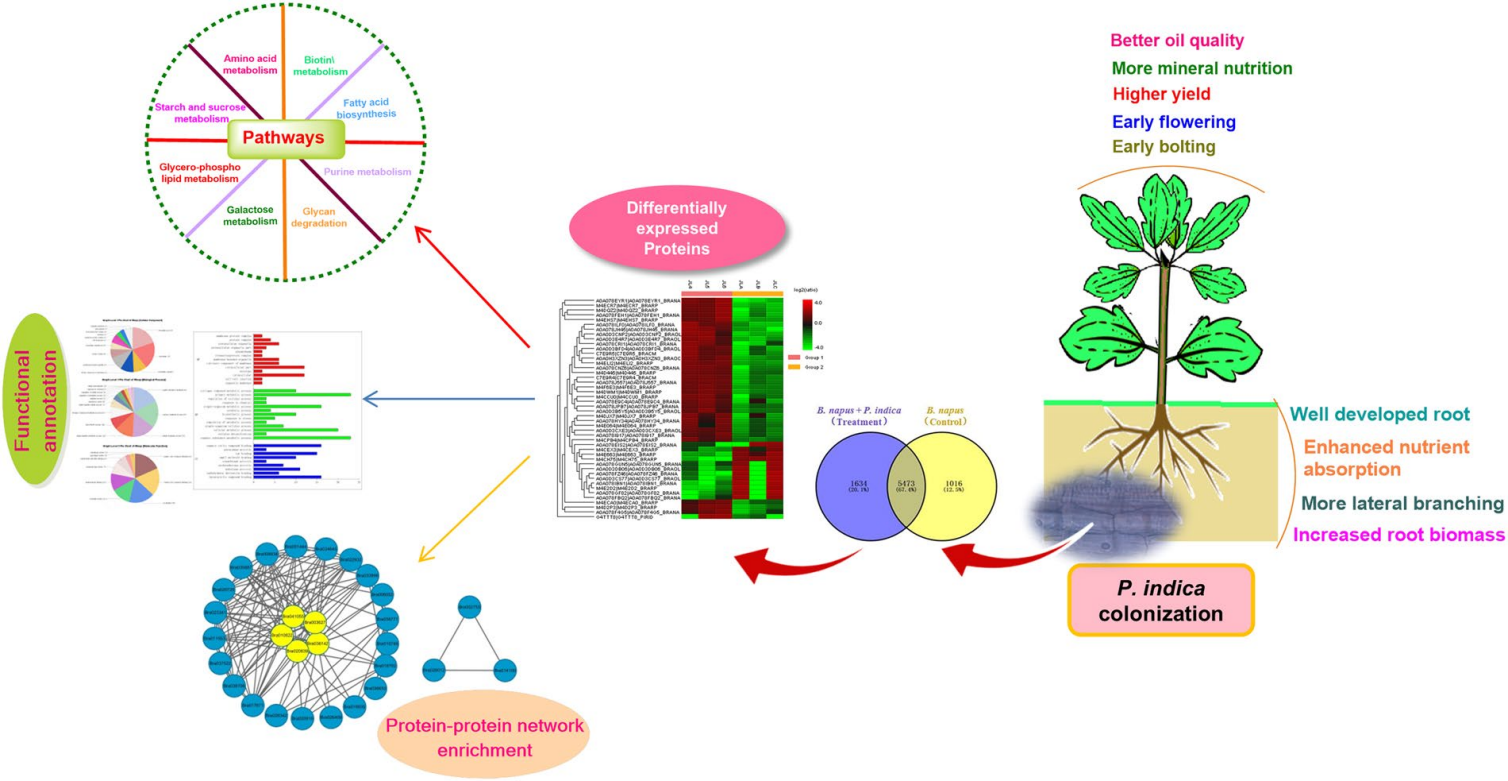
Schematic experimental approach resulted during symbiotic interaction of *P. indica* and *B. napus*.

The results of the quantitative proteomics suggested that *P. indica* significantly accumulated various metabolic proteins potentially involved in metabolic pathways which lead to growth promotion and better crop productivity. We also proposed the model for early symbiotic signaling mechanism of *P. indica* colonized rapeseed plant at the early symbiotic stage. The physiological and biochemical proteome network illustrated in our study supports the complex proteomic status of plant-fungal interaction. This study also provides the comprehensive insight which will help in the future study that ‘how fungus modulates the cellular metabolic physiology at proteome level’ resulted in ecological benefits to the host plant. Figure 1 depicts the overall schematic approach performed in symbiotic interaction to understand the molecular physiology of symbiosis.

## Results

### Root colonization and microscopic analysis

**Figure 2:**
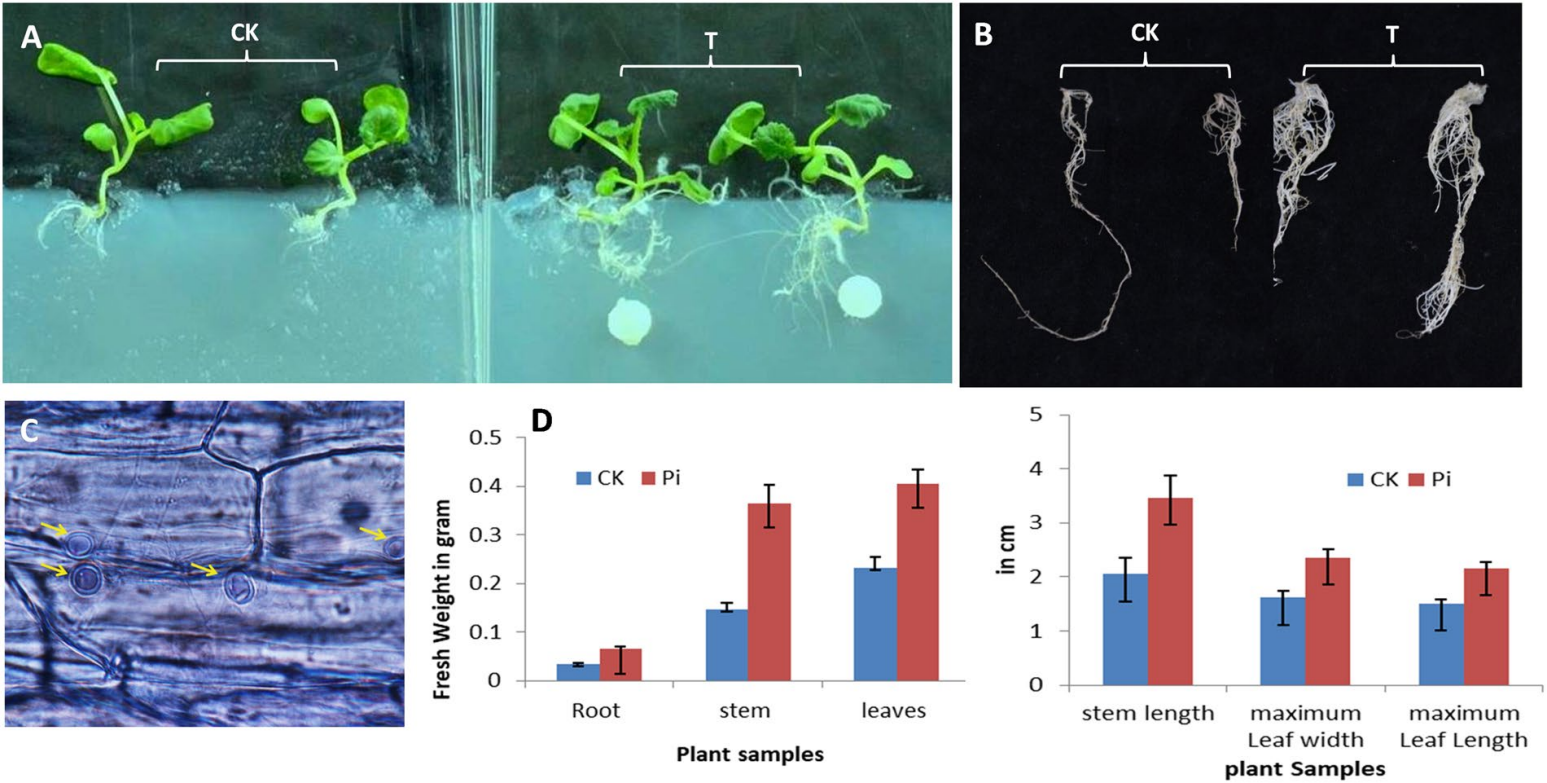
(**A**,**B**) Four weeks old *B. napus* seedling and roots, (**C**) Microscopic image of root cross section showing the chlamydospores inside the root cortex, (**D**) Agronomic parameters of treated and control *B. napus* root and shoot samples after 28 days of *P. indica* treatment. The values are the means ± SD (CK: Control; T: Treatment).

During the co-culture with *P. indica*, mycelium plugs were introduced 1 cm apart from four-leaved rapeseed plantlets. There was no physical contact with each other during the initial 2–3 days period. Vahabi *et al*.^7^ suggested, during this stage of co-cultivation, both communicate via the gaseous phase of exudate soluble compounds. After 5–6 days of the experiment, fungal mycelium and growing roots were approached to each other and penetrates into the epidermal layer of root and subsequently, sporulation started inside the root cortex and symbiosis established between both the partners. On 28^th^-day post inoculation, branched roots, more lateral rootlets with numerous root hairs were well developed in *P. indica* treated *B. napus* (Fig. 2A,B). The *P. indica* treated host rapeseed was uniformly healthy as compared to non-mycorrhizal rapeseed. At this time, the significant difference was observed in fresh weight of the root, shoot and, leaves. The length of root, shoot and leaves and leaves width were also found significantly higher in treated as compared to control plants. The microscopic study revealed the promotion of hyphal network inside the root system and penetrating the epidermal and cortical tissue^12^ (Fig. 2C). Dark colored pear shaped chlamydospores scattered around these tissues. At the later stage, these chlamydospores germinate and form the mycelium inside and finally localized as inter and intra mycelium into the host plant root.

### Quantitative proteomics of *B. napus* and *P. indica* interaction

The roots of rapeseed plant colonized with *P. indica* changed the protein profiles during the mutualistic symbiotic interaction. To assess the proteome coverage on the basis of physiological and growth parameters, we have selected 28 days old plants for the quantitative analysis. From pooled mixture of all control and *P. indica* treated root samples (in triplicate) by using LC-MS/MS method, a total of 109,003 MS/MS denovo spectra (avg. ~18,167) and a total 21,688 MS/MS peptide-Spectrum matches (avg. ~3,615) were identified and searched against the PEAKS studio 8, which corresponds to a total of 8,123 proteins at 95% confidence level. (Table 1 & Supplementary Table S1). Among, 8,123 total identified proteins, 8,067 proteins were from *Brassica* species and 56 proteins belong to *P. indica*, expressed inside the *B. napus* root.

**Table 1:** Statistical overview of proteome obtained after filtration of control (C) and treated (T) samples (in triplicates).

Sample Name	C1	C2	C3	T1	T2	T3
**Peptide-Spectrum Matches**	2864	2621	6148	2413	4276	3366
**Peptide sequences**	2307	1944	4102	1911	2919	2342
**Protein groups**	1016	931	1514	892	1229	1019
**Proteins**	2263	1865	3152	1856	2561	2306
**Proteins (#Unique Peptides)**	204 (>2); 341 (=2); 789 (=1);	113 (>2); 175 (=2); 652 (=1);	320 (>2); 497 (=2); 979 (=1);	119 (>2); 229 (=2); 621 (=1);	177 (>2); 357 (=2); 812 (=1);	201 (>2); 372 (=2); 759 (=1);
**FDR (Peptide-Spectrum Matches)**	8.60%	10.30%	3.10%	6.10%	5.60%	4.70%
**FDR (Peptide Sequences)**	10.50%	13.60%	4.40%	7.50%	8.20%	6.40%
**FDR (Protein)**	3.40%	4.30%	4.50%	4.00%	4.70%	2.30%
**De Novo Only Spectra**	16583	22854	17335	15753	21321	15157

**Figure 3:**
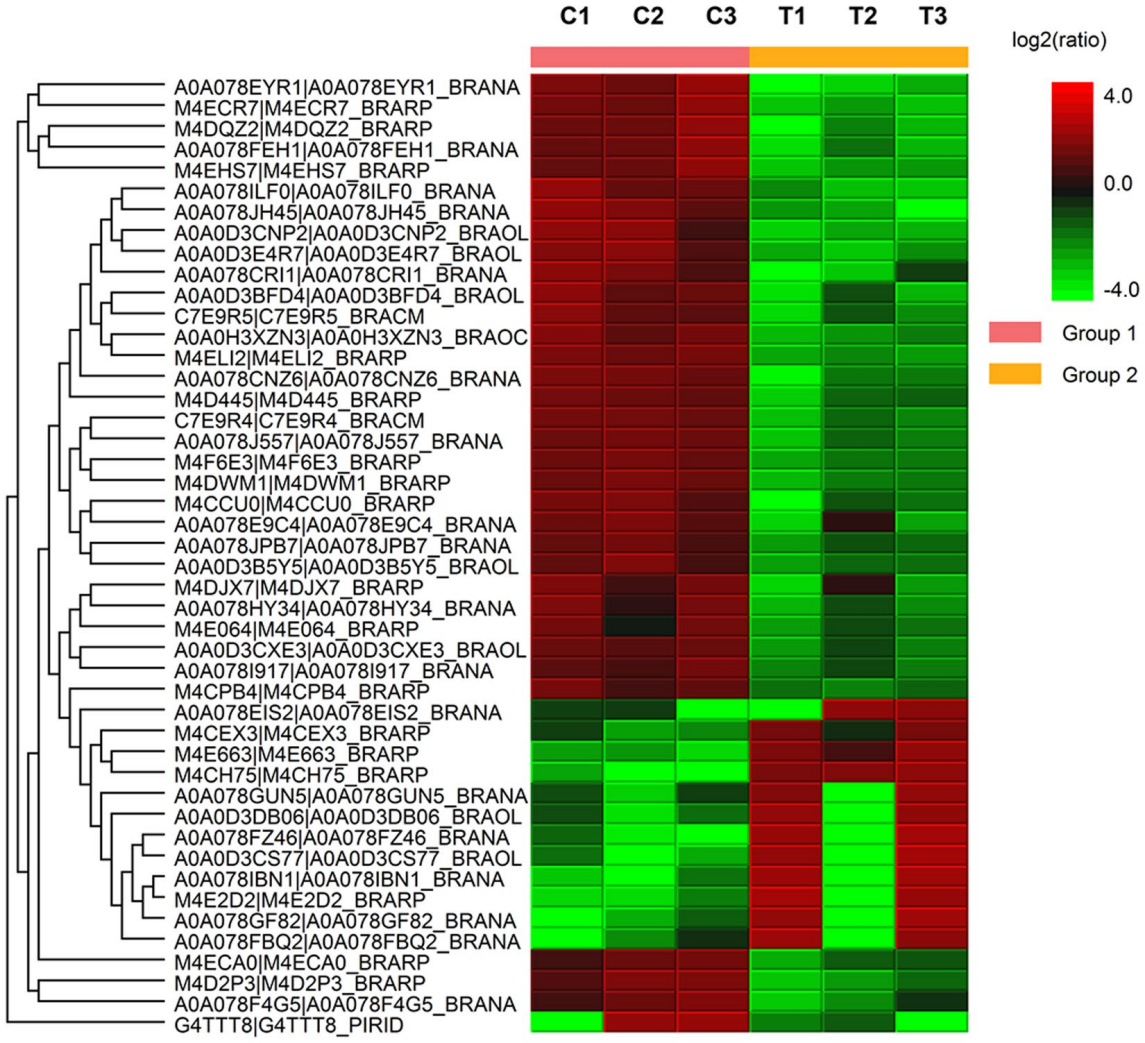
The heatmap showing the expression profiles and hierarchical clustering of 46 differentially expressed proteins (DEPs) in *P. indica* colonized *B. napus* plants. (C-control group and T-treated with *P. indica*) (Experiment performed in triplicates).

We performed the pair-wise comparison of all samples of control and treatments in triplicate to explore the similarities and differences in the symbiotic root proteome of *B. napus* plants. By using label-free quantification with following the parameters such as 95% confidence level (p-value ≤ 0.05), fold change ≥2, at least one unique peptide as a cut offs with significant method ANOVA, we identified fungal and plant proteins which are differentially expressed after efficient colonization of *P. indica* in rapeseed plant’s root. As a result, we found 46 proteins are differentially expressed in untreated control and treated plant roots (Table 2, Supplementary Table S2, and File S1). Among 46 proteins, 45 were plant proteins and 01 was fungal protein - *P. indica* (expressed inside the host plant root). Of these, 12 proteins were upregulated and 34 proteins were downregulated. Hierarchical clustering showing differentially expressed proteins in two groups of each treatment indicates that root colonization by *P. indica* induced large changes in protein levels (Fig. 3). To improve the identification of unknown proteins obtained in our result, we performed BLAST analysis against the *Brassica* species using NCBI database.

**Table 2:** Properties of differentially expressed proteins.

UniProt accession	Protein name and species	Significance (−10lgP*)	Sequence Coverage (%)	Peptide	Unique Peptide	Fold change (ratio of T/C)**	Expression
M4ELI2	Uncharacterized protein *(Brassica rapa)*	35.08	17	2	2	0.27	Down
M4CH75	Uncharacterized protein (*B. rapa*)	33.24	37	14	2	13.39	Up
M4F6E3	Acid beta-fructofuranosidase 3, vacuolar-like (*B. rapa*)	28.38	28	13	8	0.34	Down
M4DWM1	Reticuline oxidase-like protein (*B. rapa*)	26.63	10	6	3	0.31	Down
A0A0H3XZN3	ATP synthase subunit alpha, chloroplastic OS (*Brassica oleracea*)	21.78	13	5	5	0.27	Down
M4D2P3	Niemann-Pick C1 protein (*B. napus*)	20.18	4	4	4	0.28	Down
A0A078CNZ6	Alpha-mannosidase OS (*B. napus*)	19.8	36	29	1	0.27	Down
C7E9R4	Peroxidase 12(Fragment) OS (*B. rapa*)	19.8	22	6	6	0.33	Down
A0A0D3DB06	Pro-resilin-like (*B. oleracea*)	18.85	9	4	1	2.66	Up
A0A078FEH1	Germin-like protein subfamily 2 member 4 (*B. napus*)	18.53	17	3	2	0.21	Down
M4DQZ2	LRR receptor-like protein kinase At1g51890 (*B. rapa*)	18.47	3	2	2	0.17	Down
G4TTT8	Related to cysteine synthase (*P. indica*)	18.15	5	1	1	0.29	Down
A0A078JPB7	Alpha-amylase/subtilisin inhibitor-like (*B. napus*)	18.02	20	3	3	0.47	Down
M4ECR7	Basic blue protein (*B. napus*)	17.73	39	3	3	0.16	Down
A0A078EYR1	Adenylosuccinatesynthetase (*B. napus*)	17.4	18	6	2	0.1	Down
A0A078I917	RNA-binding protein 8A-B-like (*B. napus*)	17.17	37	6	3	0.5	Down
A0A078J557	Epidermis-specific secreted glycoprotein EP1-like (*B. napus*)	17.13	26	12	1	0.34	Down
A0A0D3CXE3	MLP-like protein 34 (*B. oleracea*)	17.03	61	11	3	0.45	Down
A0A0D3BFD4	Alcohol dehydrogenase (Fragment) OS (*B. oleracea*)	16.96	8	4	1	0.26	Down
A0A078GUN5	Translationally-controlled tumor protein homolog (*B. napus*)	16.87	39	7	1	3.16	Up
A0A0D3E4R7	3-oxoacyl-[acyl-carrier-protein] synthase III (FabB), chloroplastic-like (*B. oleracea*)	16.19	15	4	4	0.2	Down
M4CCU0	LysM domain-containing GPI-anchored protein 2-like (*B. rapa*)	16.04	12	3	2	0.38	Down
M4ECA0	Glycerophosphodiester phosphodiesterase GDPDL3 (*B. rapa*)	15.83	11	6	2	0.46	Down
C7E9R5	Peroxidase 21 isofrom X2 (Fragment) (*B. napus*)	15.76	18	3	3	0.31	Down
A0A078GF82	Peptidylprolyl isomerase (*B. napus*)	15.74	8	1	1	4.63	Up
M4E2D2	NADH dehydrogenase [ubiquinone] 1 alpha subcomplex subunit 13-B-like (*B. rapa*)	15.73	13	1	1	7.35	Up
M4EHS7	Receptor-like kinase TMK4 (*B. rapa*)	15.54	7	4	2	0.21	Down
A0A078CRI1	Nudix hydrolase 3-like (*B. napus*)	15.42	7	4	4	0.2	Down
A0A078ILF0	N-acetyl-gamma-glutamyl-phosphate reductase (*B. napus*)	15.16	23	7	7	0.18	Down
M4CPB4	3-hydroxyacyl-[acyl-carrier-protein] dehydratase FabZ-like (*B. rapa*)	15.05	27	6	5	0.48	Down
A0A078JH45	Eukaryotic translation initiation factor 3 subunit F OS (*B. napus*)	15.03	25	6	2	0.15	Down
A0A0D3B5Y5	Eukaryotic translation initiation factor 3 subunit F (*B. oleracea*)	14.98	32	7	3	0.41	Down
A0A078HY34	Suppressor protein STM1-like (*B. napus*)	14.92	16	3	3	0.37	Down
M4D445	Glucan endo-1,3-beta-glucosidase (*B. rapa*)	14.9	2	1	1	0.38	Down
A0A078EIS2	Uncharacterized protein OS (*B. napus*)	14.55	3	1	1	2.28	Up
A0A078FZ46	Subtilisin-like protease SBT2.5 (*B. napus*)	14.27	2	1	1	6.46	Up
M4E064	Jacalin-related lectin 35 (*B. rapa*)	14.26	34	8	6	0.49	Down
A0A078F4G5	Pectin acetylesterase 5-like (*B. napus*)	14.15	7	2	2	0.37	Down
M4E663	Peroxidase 69 (*B. rapa*)	14.1	25	8	4	5.34	Up
A0A078IBN1	RNA-binding protein CP29B, chloroplastic-like (*B. napus*)	13.92	21	5	1	6.26	Up
A0A0D3CS77	26 S protease regulatory subunit 7 homolog A-like (*B. oleracea*)	13.83	1	1	1	5.13	Up
A0A0D3CNP2	Aminoacylase-1 (*B. oleracea*)	13.83	15	6	2	0.15	Down
A0A078FBQ2	Protein modifier of SNC1 11-like (*B. napus*)	13.65	14	2	2	3.13	Up
M4CEX3	DNA ligase (*B. rapa*)	13.54	1	1	1	2.35	Up
A0A078E9C4	Monocopper oxidase-like protein SKU5 (*B. napus*)	13.38	21	10	1	0.35	Down
M4DJX7	Aspartic proteinase A1-like = GN (*B. oleracea*)	13.13	34	14	1	0.36	Down

*Low scoring peptide identifications are filtered out by setting −10lgP threshold >13 (Peak studio 8.0). **Proteins which contains the T/C (Treated/Control) ratio ≥2.28 and ≤0.5 were considered as significant.

### Functional classification of differentially expressed protein

**Figure 4:**
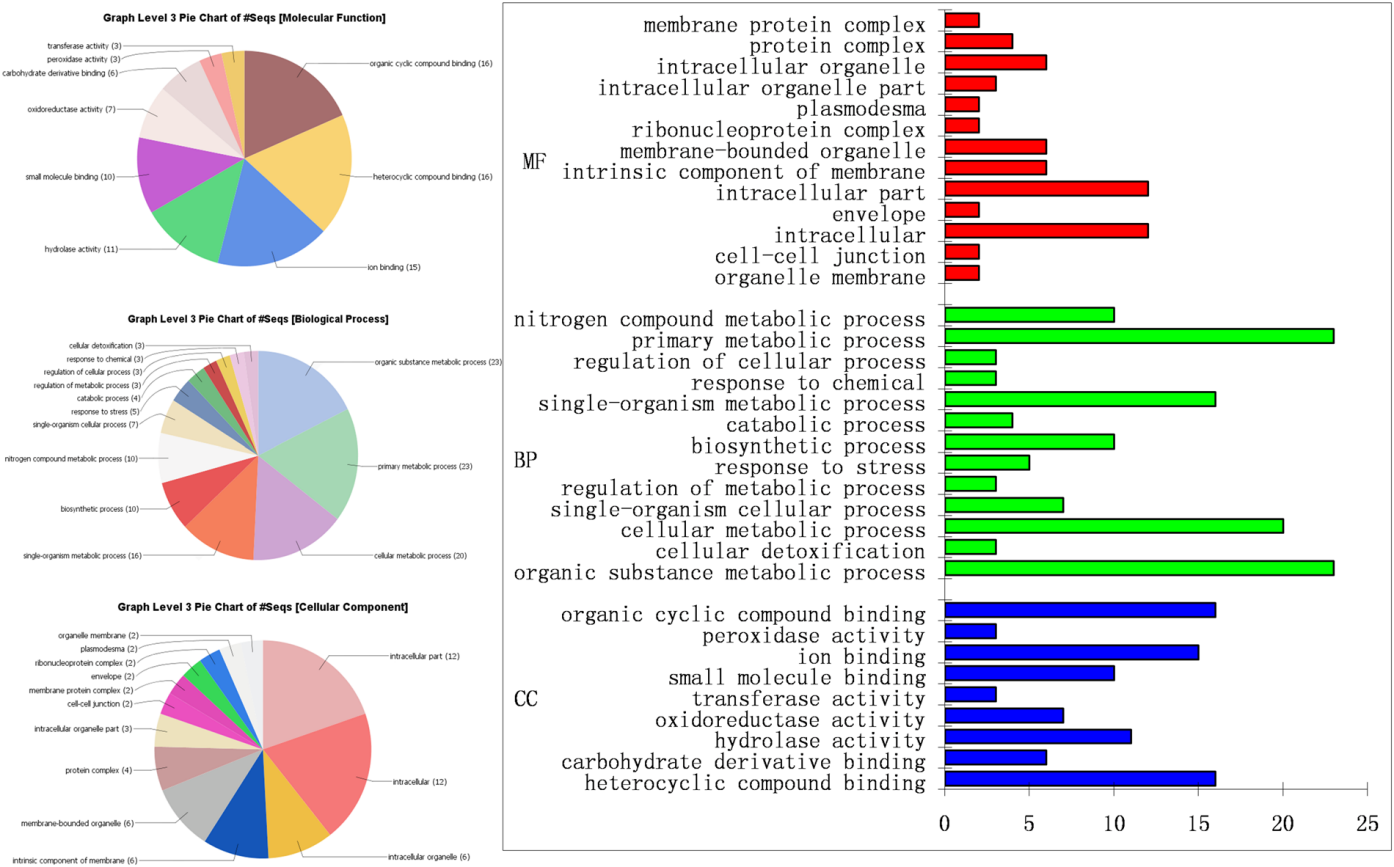
Gene ontology enrichment analysis of differentially expressed proteins accumulated in *P. indica* inhabited *B. napus* root.

In order to obtain a Gene Ontology (GO) classification for the identified differentially expressed proteins of *B. napus* plant, a GO tool blast2GO was used. The 46 proteins from *P. indica* colonized *B. napus* root are attributed to their biological process and found that 17.70% (23) proteins engaged in the primary metabolic process and 15.38% (20) involved in cellular metabolic processes. The major proportions of 17.70% (23) proteins were found to be involved in various organic substances metabolism which are essential components of biosynthesis, degradation, and energy-related reactions. 7.69% (10) proteins are involved in the biosynthetic process and the same number of proteins is also engaged in nitrogenous compound metabolism. Remaining proteins are related to various other biological regulation processes as cellular detoxification, stress responses, and regulation of catabolic, metabolic and cellular activities. Among the 46 differentially expressed proteins 29.27% (12) proteins were proposed to be located in the cytoplasm and another 17.03% (7) belonged to cytoplasmic part. However, 14.63% (6) proteins were the part of an integral component of the membrane and the same percentage was present in intracellular membrane-bound organelle. Mitochondrial proteins including membrane occupied 9.76% (4) of total identified proteins. The root proteomes of rapeseed showed overrepresented molecular functions in activities of binding like organic compound, nucleic acid, cation-anion binding, nucleotide and nucleoside binding, hydrolase, oxidoreductase activity, peptidase activity. The majority of proposed proteins under molecular function category included phosphate binding (10), nucleotide binding (10), ionic binding (16), oxidation-reduction reactions (3) and various catalytic activities (3). Figure 4 and Supplementary Table S3 represented the identified root proteins modulated by *P. indica* cover a wide range of cellular components, molecular functions, and biological processes. These biological pathways are also mapped in KEGG and were annotated in 10 KEGG pathways (Supplementary Table S5).

### Protein–protein interaction (PPI) analysis of AM fungus treated rapeseed plant

**Figure 5:**
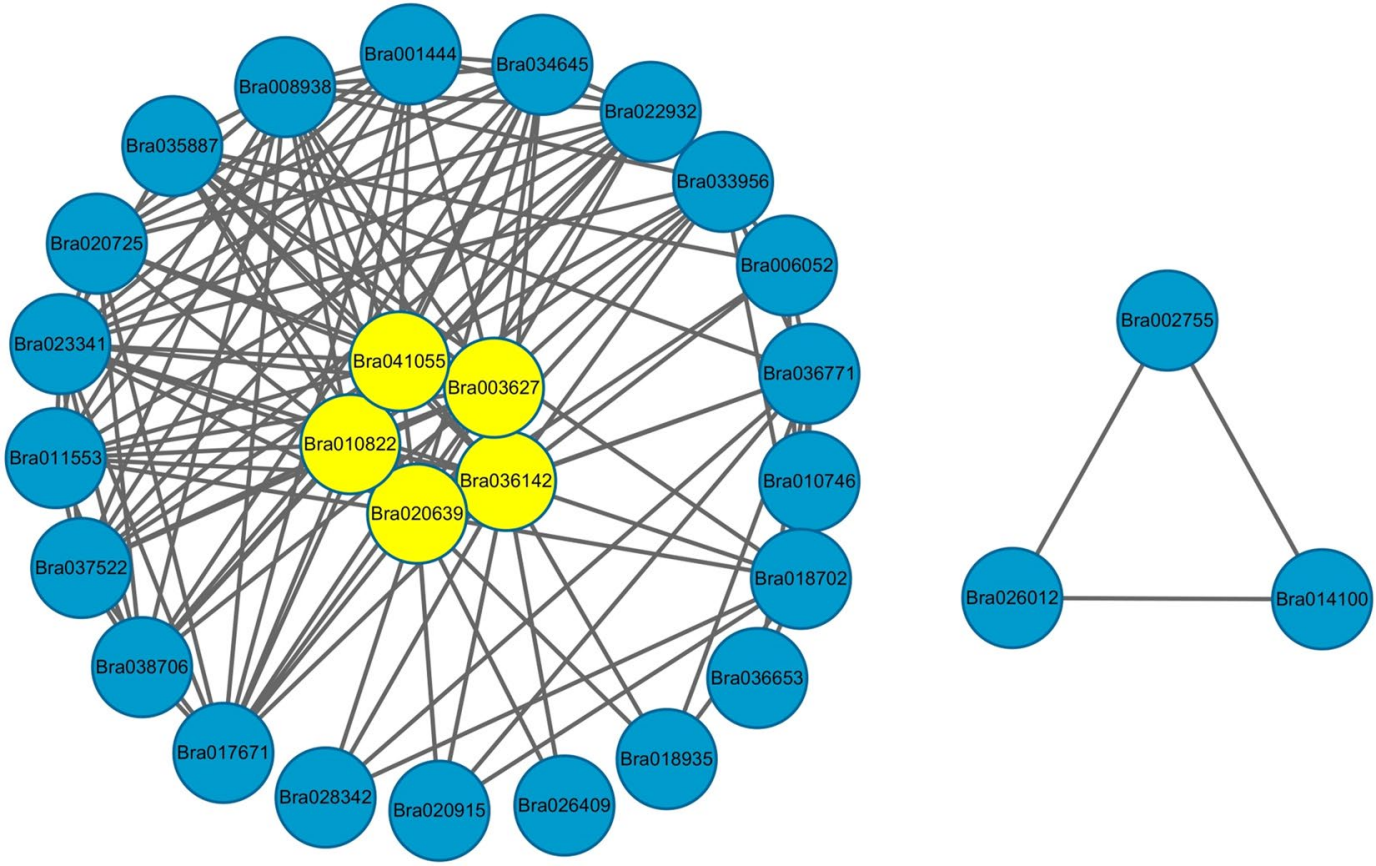
Protein-protein interaction networks of differentially expressed proteins involved in energy metabolism and protein regulations.

To explore the PPI information in the context of symbiosis responsive proteins, we analyzed the functional overview of 46 DEPs using Cytoscape v2.8.3 software. The Fig. 5 and Supplementary Table S4 depict the densely connected network, in which 16 out of 46 identified proteins were mapped to the *B. napus* protein-interactome database. The proteins which were highlighted in the network with the most connect degree were considered as drive proteins and these proteins centrally involve in energy metabolism that participate in activating, suppressing, regulating or catalyzing the cellular processes within the cell (Fig. 5). In the network, we speculated that the yellow subunits forms the interactome cluster with the other proteins and potentially drives the energy metabolic process in host plant. Another triangle interaction network is related to DNA/protein binding proteins and participates in protein regulation. The findings were consistent with our GO enrichment and KEGG pathway analysis (Fig. 3 and Supplementary Table S5).

## Discussion

In our previous study, we demonstrated the positive interaction of *P. indica* with *B. napus* in overall growth, yield, quality and nutritional attributes^12^. Further, to evaluate the proteomic changes during the multistep colonization process, relative changes in functional proteins has been assessed. We compared the treated and control plants in triplicates, resulted in identification of 46 significant DEPs in which 14 were increased and 32 were decreased. The previous gene expression studies demonstrated that, defense genes, signaling molecules, and transcription factors are upregulated during early stages of root colonization and down regulated gradually at later stages^6,7,10,18^. We speculate that, in our study, the colonization has been established during the harvesting time followed by enhanced plant growth, so this could be a possible reason of downregulation of 34 DEPs in proteomic analysis. Further, expression of DEPs finally leads to change in cellular morphology, physiological and metabolic conditions of host plant to establish successful symbiosis. Furthermore, functional annotation results in current proteomic approach with GO, KEGG and PPI network revealed the complex functions of proteomic-interactome in host-symbiont relationship (Figs 4 and 5). For instance, KEGG pathway analysis suggested a cluster of proteins which involves in regulation of cellular & biosynthetic process of carbohydrate, lipid and proteins along with metabolic regulation of nitrogenous compound and organic substances (Supplementary Table S5). In addition, functions of identified DEPs were further investigated to reveal the key factors participated in symbiotic associations.

### DEPs involved in Defense Pathway

Our proteomic data revealed the elevated level of DEPs involved in the defense/stress/cellular detoxification related proteins including three types of peroxidases [12 (C7E9R4), 21(C7E9R5) and 69 (M4E663) isoforms), germin-like protein (GLP) subfamily 2 (A0A078FEH1), epidermis-specific secreted glycoprotein EP1 (A0A078J557), MLP-like protein (A0A0D3CXE3) and jacalin-related lectin 35 (M4E064) in roots upon *P. indica* treatment. The peroxidases belong to the class III secretary plant peroxidases, reside in plant vacuoles or intracellular spaces and involved in auxin catabolism, lignin synthesis, H_2_O_2_ detoxification and stress responses^19^. The GLP subfamily member is also participated in defense and is associated with various enzymatic activities comprising oxalate oxidase (OxO), superoxide dismutase (SOD), glucose pyrophosphatase/phosphodiesterase (AGPPase), and serine protease inhibitors^20^. Song *et al*.^13^ suggested the role of lectin and GLP in defense during the interaction of AMF *G. mosseae* and *A. fruticose*. Additionally, EP1 and lectins are involved in carbohydrate recognition and glucan binding and play an important role during pathogen attack and maintain the cell integrity^21,22^. Earlier events suggest that initially root recognizes arbuscular mycorrhizal (AM) fungus as a pathogen and trigger weak defense responses^6,7^. At this phase, defense genes are expressed and reactive oxygen species (ROS) produced by the host against *P. indica*^11^. Zuccaro *et al*.^23^ studied the *P. indica* gene expression during colonization of barley roots and reported the elevated level of pre-penetration phase genes engaged in oxidative stress, flavonoid-phenolic compounds reduction, and extracellular dioxygenase activity and in advance stage of colonization stress-related genes, cytochrome P450, glutathione S-transferase, quinine-reductases were found elevated. *P. indica* exhibits biotrophic colonization strategy, which includes host’s cell death-associated colonization phase, reported in *Arabidopsis* and barley^23–25^. We hypothesized that these differentially expressed stress proteins accumulated in cell-death colonization phase which help the colonized plant to maintain the cell homeostasis and enhanced plant resistance from oxidative, biotic, edaphic and environmental stress during various growth and development stages.

### DEPs related to pre-symbiotic and cellular signaling

The onset of symbiosis requires perception of chitin-based molecules (lipo)-chitooligosaccharides (LCO), short-chain lipochitooligosaccharides Myc-LCO, and short chitooligosaccharides (CO_4_) as putative symbiotic signal molecules derived from AM fungus, which is very similar to rhizobial NFs^26,27^. During the invasion of a foreign organism, a signaling cascade of plasma membrane-localized receptor complexes governs the mutualism through the variety of signaling molecules at root-soil interface. The signaling pathway involves various signal transducers to start molecular dialogue between both the partners like; lysine motif (LysM) domain carrying proteins, and leucine-rich-repeat receptor-like kinases (LRR-RLKs)^27–30^.

In *P. indica* colonized root of *B. napus*, we identified the LysM domain-containing GPI-anchored protein (M4CCU0), which corresponds to the chitin perception mechanism reported in rice^30,31^. During the onset of symbiosis, Myc-LCO/CO4/chitin is perceived by two plasma membrane receptor; CEBiP (Chitin elicitor binding protein) and CERK1 (Chitin elicitor receptor kinase) with LysM^28,29^ (Supplementary Fig. S1). Zipfel *et al*.^29^ suggested that successful colonization depends on the coordinated perception of Myc-LCO and Myc-chitin oligosaccharides in AM symbiosis^29^. LysM RLK RNAi knockdown experiments suggest the role of Myc–COs and LysM RLK during the initial AM fungal-host signaling in rice, legume and dicot non-legume plants^27,32^.

Here, we witnessed another protein, LRR receptor-like protein kinase At1g51890 (M4DQZ2), which enables to recognize fungal chitin, small peptide molecules and also proteins^33^ (Supplementary Fig. S1). Plant LRR-RLKs possess a functional cytoplasmic kinase domain to perform ser/thr kinase activity^34^. Previously, plasma membrane-localized LRR and MATH domain-containing proteins were also reported in *P. indica* symbiosis outcome with growth promotion^24,35^. Shahallori *et al*.^36^ reported a LRR protein named *pii-2* in *Arabidopsis*, responsible for *P. indica-*mediated growth promotion and enhanced seed production.

Recognition of microbe-associated molecular patterns (MAPS) by host plants accelerates the downstream transmission of signals, which activate other receptors/proteins to regulate the series of cellular events. This leads to notable transcriptional changes to regulate the expression of symbiotic genes. These events mediated by the nuclear calcium oscillations in response to Myc-LCO, which is considered as a hallmark of symbiotic responses^29,30^. Our data contain following proteins related to calcium signaling; subtilisin-like protease SBT2.5 (A0A078FZ46), (significantly accumulated), calcium binding EF-hand family protein (L7PBC3), and calmodulin (CaM) related (Q710C9) (non-significant, Supplementary Table S1 - Total proteins). SBTs engaged in AM symbiosis and help the plant in ‘sym’ Pathway to perceive the LCOs signal generated by endosymbiotic fungus. This spawns the signaling cascade in root epidermis and subsequently develops the pre-penetration apparatus which lead to infecting the host tissue by arbuscules formation. Further, subtilase genes restructure the plasma membrane^26,37^.

Our findings support the role of LRR, LysM, calcium binding EF-hand family protein, SBTs, and CaM, mediated signaling in *P. indica* colonized *B. napus*. Supplementary Fig. S1 showed the proposed model of signaling during establishment of symbiosis between the plant and fungal partners.

### DEPs in Cell architecture and cell wall remodeling

In this study, we also found significantly expressed proteins like receptor-like kinase TMK4 (M4EHS7), pectin acetylesterase (A0A078F4G5), monocopper oxidase [SKU5, a glycoprotein with GPI anchor (A0A078E9C4)] and reticuline oxidase (M4DWM1), responsible for the structural changes during endophytic colonization. The TMK subfamily acts in a functionally redundant manner and interacts with Auxin binding protein 1 (ABP1) to regulate ROP (Rho of Plant) signaling. This critically controls the cell expansion, division, proliferation and downstream of auxin at the cell surface^38,39^. The enzyme pectin acetylesterase is responsible for O-acetylation of pectin, which plays important role in maintaining cell wall architecture during growth and differentiation of plant tissues^40^. Similarly, monocopper oxidase regulates the multiple-copper oxidases which participate in directional root cell wall development and growth^41,42^. Another protein reticuline oxidase, a berberine bridge enzyme is also reported to be involved in the modification of cell wall architecture during growth and it also responds against the pathogen^43^. These signaling events and activation of cellular architecture proteins help the tissue to reorganize the structure which further helps fungi to develop a highly branched arbuscules inside the cortical cells. These arbuscules surrounded by plasma membrane-derived peri-arbuscular membrane localized various protein moieties which principally take part in nutrient acquisition^30,32,44^.

### Proteins related to lipid, nitrogen and phosphorous metabolism

**Figure 6:**
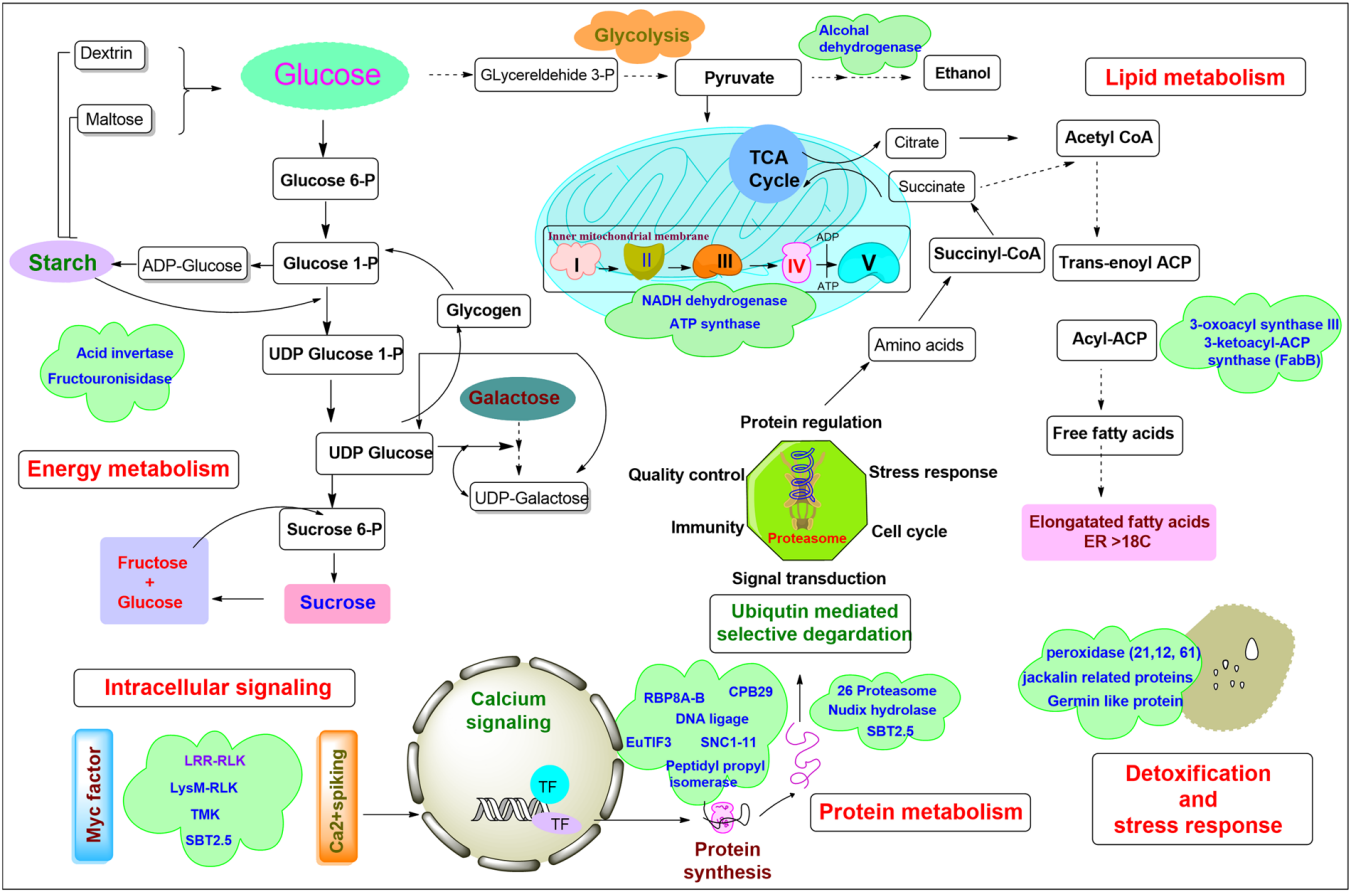
Schematic illustration of differentially expressed proteins (DEPs) associated with major metabolic protein networks during symbiotic association of *P. indica* and rapeseed plant. DEPs that have been significantly detected at the proteomics level were highlighted in green colored clouds.

In plants, fatty acids and lipids are the building blocks of membranes and also required for the senescence, tolerance against cold temperatures and phosphate starvation which helps plants to perform better^45,46^. Here, two DEPs, 3-oxoacyl-[acyl-carrier-protein] (FabB) synthase III (A0A0D3E4R7) and 3-hydroxyacyl-[acyl-carrier-protein] dehydratase (FabZ) (M4CPB4) were significantly accumulated in *P. indica* colonized root (Fig. 6). The KEGG pathway study showed the role of FabB and FabZ as an intermediate candidate in metabolic flux distribution of fatty acid biosynthesis pathway (Supplementary Table S5). FabB-condensing enzyme along with other members is responsible to elongate the chain of saturated and unsaturated fatty acids in C_12_–C_16_ range of chain lengths and control the level of unsaturated fatty acids^47^. However, the FabZ protein is involved in dehydratase catalytic activity during the lipid A biosynthesis. Both DEPs take part in fatty acid elongation, degradation, and metabolism of glycerol-lipid, glycerol-phospholipid and lipoic acid that helps in providing raw materials to newly synthesized cells during root colonization^47^.

Nitrogen (N) and phosphorous (P) metabolism are prominence factor in plant’s growth and development. N-acetyl-gamma-glutamyl-phosphate reductase (A0A078ILF0), aminoacylase (A0A0D3CNP2), and adenylosuccinate synthetase (A0A078EYR1), significantly expressed in this study, are key intermediate enzymes in arginine biosynthesis pathway and purine, arginine, glutamate, aspartate metabolic pathway, respectively (KEGG pathway, Supplementary Table S5). Upregulation of these pathways supports the enhanced nitrogen and phosphorous assimilation by *P. indica* in the colonized host plant. A similar study was performed by Govindarajulu*et al*.^48^ in AM symbiosis and concluded that nitrogen transferred from fungus as ammonium, is used in the synthesis of arginine which further broken down in ammonium and takes part in amino acid biosynthesis.^49,50^.

Previously, *P. indica* is reported to enhance phosphorous metabolic enzymes which contribute in higher phosphate uptake in plants^44^. In this study, enzyme glycero-phosphodiester phosphodiesterase GDPDL3 (M4ECA0) is significantly accumulated and helps in catalysis of phospholipids to break down into phosphodiesters, which further lead to release phosphorous by various intermediate steps^51^. Enhanced arginine metabolism and binding ability of arginine with polyphosphate also suggest its role in translocation of fungal phosphorous to host plants^49^. These events advocate that *P. indica* colonization possibly increase the uptake of phosphorous acquisition. In our previous study, we also reported ~17.58% & ~23.34% more nitrogen accumulation and 25.68% & 30.83% more phosphorous accumulation in *B. napus* leaves and seeds respectively, as compared to control^12^.

### DEPs in Proteins synthesis and fate

During symbiosis, the RNA binding protein CPB 29B (A0A078IBN1), RNA binding motif protein 8A-B (RBM8A-B) (A0A078I917) and Peptidyl-prolyl isomerase (A0A078GF82) were found significantly accumulated, which take part in protein synthesis and folding (Fig. 6). The CPB 29B is a member of small family cpRNPs which are engaged in RNA regulatory functions and reported in tobacco, associated to RNA stabilization. The cpRNPs bind to target internal sequences of endoribonucleases and protect it from degradation^52^. RBM8A together with Magoh, associated with decapping, methylosome activity, and centrosome maturation, and their deficiency results in G2/M accumulation, followed by cell apoptosis^53^. Nevertheless, Peptidyl-prolyl isomerases are enzymes that are responsible for protein folding by cis-trans isomerization of X-Pro peptide bonds and also bind to mature proteins to control their activity, subunit assembly and localization^54,55^. Other significantly accumulated DEPs are eukaryotic translation initiation factor 3 (A0A078JH45), protein modifier of SNC1 11 (A0A078FBQ2) and DNA ligase 6 (M4CEX3), are important components of cellular protein synthesis and regulation.

During the symbiosis process, the cell metabolism is critically regulated by the periodical synthesis, modifications, degradation of peptides & proteins and programmed cell death. In our root proteome results, 26S proteasome (A0A0D3CS77), nudix hydrolase (A0A078CRI1), suppressor protein STM1-like (A0A078HY34), subtilisin-like protease SBT2.5 (A0A078FZ46) were found significantly upregulated (Fig. 6). From embryogenesis to seed shredding, majority phases of plant life require the selective degradation of regulatory proteins, which is tightly regulated by the ubiquitin-26S proteasome system. Apart from selective degradation, 26 proteasome helps to clean unwanted and abnormal intracellular proteins of cytoplasmic and nuclear origin via ubiquitin/26S proteasome pathway to maintain cellular homeostasis during initial and advance stages of symbiosis. It is also engaged in protein trafficking, site-specific proteolysis and limiting the protein activity^56–58^.

Another accumulated important enzyme in our experiment was subtilisin-like protease SBT2.5, which utilizes the storage protein and is a key component to create plant architecture. It critically associated with whole plant cycle; including germination, vegetative and reproductive growth, embryo, flower, seed development to till senescence. SBTs play a crucial role in peptide hormone synthesis, and also contribute to signal biogenesis in plants^26,58,59^. Protein family nudix hydrolase is a small protein (16–21 kDa), known for cellular ‘house cleaning’ activity. It is engaged in RNA processing, the directive of ERK signaling, calcium channel, the breakdown of various deleterious endogenous metabolites including, a wide range of nucleoside di- and triphosphate derivatives, nucleotide sugars, and a various substrates specific RNA caps^48,60^. Fungal intervention with the host increases the metabolic activity for rapid growth, which produces lots of deleterious metabolites as a by-product of metabolic reactions. The above-explained proteins identified in our study supports our hypothesis that these are the key proteins which help to maintain the cellular homeostasis by house-cleaning activity of the host plant. PPI network also depicted the interactome network of DNA binding proteins participates in protein regulation during various biological process (triangle network in Fig. 4).

### Proteins engaged in energy synthesis

PPI network reveals that the 16 proteins with 5 driver proteins centrally involved in energy metabolism (Fig. 5). These proteins regulate the carbohydrate metabolism and energy synthesis pathway to maintain energy need during symbiotic growth. Among the DEPs related to carbohydrate metabolism, acid β-fructofuranosidase (M4F6E3) is a key intermediate of galactose, starch and sucrose metabolism (KEGG pathway) and takes part in cell wall biosynthesis (Fig. 6 & Supplementary Table S5). Additionally, enzyme alcohol dehydrogenase (ADH) (A0A0D3BFD4) is also an important component of carbohydrate metabolism and required for cell division and elongation^61^.

During the symbiosis, the NADH dehydrogenase (ubiquinone) 1 alpha (M4E2D2), peptidylprolyl isomerase and ATP synthase (Complex V) (A0A0H3XZN3) were significantly accumulated in colonized rapeseed root, possibly played role in the enhanced growth and developmental process. The NADH dehydrogenase (oxidoreductase complex I) participates in electron transfers from NADH to ubiquinone in ETC and at the same time involved in proton translocation across the mitochondrial membrane. Subsequently passing from the various complexes I-IV, as a result, a proton gradient is formed inside the mitochondrial membrane and further this is used by the ATP synthase complex (complex V)^62,63^. The ATP synthase (the final enzyme in oxidative phosphorylation pathway) binds with mitochondrial F^0^ and F^1^ regions and catalyzes the terminal step of ATP formation using electrochemical gradient generated across the membrane by protons during oxidative respiration^62–64^. The membrane ATP synthase was studied in hosts *Medicago truncatula* and *Pteris vittata*, colonized by AM fungus *Glomus intraradices*^65,66^. Two fungal mitochondrial ATP synthase along with membrane ATPase activity was also detected by Becard *et al*.^67^ in pre-symbiotic phase suggested the role in energy-dependent functions in AM colonized host roots^66,67^. This study further supports our current host-symbiont proteome results.

ATP production is highly regulated by energy needs, thus the increase in ATP production suggests intense energy demand in the root of rapeseed that could coincide with the switch from asymbiotic to pre-symbiotic growth phase. Accumulation of energy-related DEPs suggests the energy required to maintain both the symbiotic partner together. In later stages, plant growth is enhanced and also the more intense hyphal branching of *P. indica* inside the root was observed.

### Fungal DEPs and unknown proteins

Here, we identified total 56 proteins from *P. indica* expressed inside the *B. napus* root (Supplementary Table S1). Out of which, cysteine synthase (G4TTT8) enzyme was found significantly accumulated by label-free proteomics. During the initial colonization phase, reducing environment generates inside the fungus. Cysteine-containing molecules glutathione and thioredoxin protect the fungus against oxidative stress generated by reducing environment^68^. Out of 46 DEPs, three new symbiotic proteins from colonized rapeseed plant were appeared with unknown function and will be targeted for future study to understand the further role in symbiotic associations.

Figure 6 represents the tentative mapping of symbiotic proteins identified to major pathways of intercellular signaling, glucose and lipid metabolism, energy synthesis and protein regulation (synthesis, selective degradation, and protein lysis). These metabolic pathways are interconnected with each other and key sources of plant growth and development. The structure drawn is based on knowledge available from plants metabolic pathways. The represented proteins/enzymes span key steps of the various regulatory pathways modulated during symbiotic association of *P. indica* and *B. napus*.

In conclusion, the 46 DEPs identified in our study are the part of complex cellular and molecular processes in symbiotic association between host and fungus. The quantitative study of protein reveals their role in diverse physiological pathways. This study verifies the positive symbiosis between the host *B. napus* and AM fungi *P. indica*. Exploring these protein functions, our study provides a comprehensive understanding of root’s architectural modification, cell remodeling and maintenance of cellular homeostasis during the symbiotic and growth phase of plant’s life. Finally our study provided new candidate proteins to carry out further study on this beneficial association of plant-fungal interaction. These findings will further help the researchers to understand the molecular networks and modulation of biological pathways behind the plant-fungal interaction extensively.

## Material and Methods

### Fungus

The plant growth promoting fungus *P. indica* was collected from State key laboratory of Rice Biology, Institute of Biotechnology, Zhejiang University Hangzhou, China. *P. indica* was grown on modified Kafer medium^69^ at 25 °C for 5 days in the dark. The two fungal plugs (5 mm each) were picked out and inoculated into a glass petri dish (90 mm × 90 mm) which contains 50 ml of modified plant nutrient medium (PNM)^70^. The fungus was incubated at 25 °C for 7 days.

### Plant Materials

For the experiments, *B. napus* seeds were collected from State key laboratory of Rice Biology, Institute of Biotechnology, Zhejiang University Hangzhou. Before the experiments, rapeseeds were soaked in distilled water for 12 h. The seeds were surface sterilized and inoculated in Murashige and Skoog medium^71^ for germination. The four-leaved rapeseed plantlets with consistent height were selected and transferred to new MS medium plate. The fungal hypha plug of 50 mm diameter from 7 days old *P. indica*, was inoculated 1 cm apart from the root of *B. napus* seedlings. Further, these plates were transferred to plant growth chamber under growth conditions 25/22 °C (day/night), 16/8 h (alternating light and dark), with a light intensity of 5,500 lx. The samples were harvested after four weeks; leaf, root and stem were separated. The roots were washed carefully in distilled water, weighted both separately and immediately frozen in liquid nitrogen. Further, samples were stored in a −80 °C freezer until protein extraction.

### Root Colonization study

The roots of 15 days old rapeseed seedlings were washed thoroughly with tap water, cut into small pieces (1 cm) and treated with 10% KOH solution at room temperature for overnight. The roots were rinsed repeatedly using sterile distilled water, then dipped in 1% HCl solution for 1 min and then stained with 0.05% trypan blue for 3–5 min, finally rinsed several times in sterile water. The stained roots were observed under an Olympus confocal microscope BX51 (Tokyo, Japan) for *P. indica* root colonization.

### Protein extraction, digestion and desalting

To evaluate the proteins during interaction study, proteins were extracted from 28 days old *B. napus* root samples in triplicates (200 mg per plant) by trichloroacetic acid (TCA) - acetone - phenol method^72^. The protein concentration was measured by the Bradford method^73^. Further, 30 μl (approx. 100 μg) total protein was reduced with 10 mM dithiothreitol (DTT) for 30 mins at 37 °C, then alkylated with 100 mM iodoacetamide (IAA) for 20 mins at room temperature. Finally, samples were digested with 40 μl of 0.05 M ammonium bicarbonate (ABC) with trypsin (enzyme to protein ratio 1:50) and incubated overnight at 37 °C (followed according to manufacturer’s instructions - Millipore).

### Mass spectrometry analysis

A Liquid Chromatography/Mass Spectrometry (LC/MS) system consisting of a Nano-nLC 1000 system (Thermo Fisher Scientific) connected to a linear quadrupole ion trap–Orbitrap (LTQ Orbitrap Elite) mass spectrometer (Thermo Fisher Scientific) equipped with a nanoelectrospray ion source was used to analyze the tryptic peptides. Total 2 μg of peptides mixtures were separated by C18 reverse-phase chromatography on an Acclaim PepMap RSLC with a length of 15 cm, an inner diameter (i.d.) of 50 μm, and a particle size of 2 μm using a pre-column (Acclaim PepMap 100, length 2 cm, i.d., 75 μm, particle size 3 μm) and a mobile phase gradient from 3% to 8% (v/v) acetonitrile (ACN), 0.1% (v/v) formic acid (FA) in 10 min, 8% to 20% (v/v) ACN, 0.1% (v/v) FA in 15 min, 20% to 30% (v/v) ACN, 0.1% (v/v) FA in 17 min and 30% to 90% (v/v) ACN, 0.1% (v/v) FA in 8 min with a flow rate of 250 nL/min. Peptides were electro sprayed online into an LTQ-Orbitrap Elite mass spectrometer via a ProxeonNanoSpray Flex Ion Source (Thermo Fisher Scientific) at 1.8 kV (source voltage) and the temperature was set at 300 °C. Peptides were detected by full scan mass analysis from m/z 300–2,000 at resolving power of 60,000 (at m/z 400, measured at full width at half-maximum peak height (FWHM); 1-s acquisition). Collision-induced dissociation (CID) was applied as the fragmentation technique with a collision energy of 35 eV. MS/MS with rolling collision energy of the 20 most intense precursor ions with charge states 2^+^ to above.The spectral data was acquired by ThermoXcalibur version 3.0.

### Protein identification and quantification

The raw spectrum files were processed and quantitative ratio determined using PEAKS Studio version 8 (Bioinformatics Solutions) with a parent mass tolerance of 25.0 ppm, and fragment tolerance of 0.8 Da. PEAKS studio 8 was used to interrogate a custom database containing all *Piriformospora* and *Brassica* proteins on UniProt database combined with Post-translational modifications (PTMs). To assess modifications carbamidomethyl was set as fixed, oxidation, deamidation, and 482 built-in-modifications were set as variables. Maximum missed cleavage was set at 3 and false detection rate (FDR) < 1%. For the quantification we used label-free proteomics to identify the differentially expressed proteins (DEPs) with significant score (−10lgP) > 13 were used for protein acceptance. Minimum unique peptide was set at 1. Highly differentially expressed proteins between control and treatment groups were identified by statistical analysis tool (fold change > 2, FDR < 1%) and presented in heatmap format (generated by PEAKS studio 8). The ANOVA was used to calculate significance (in-build in Peak Studio 8).

### Data analysis

Plant experiments performed *in-vitro* were arranged in a completely randomized design and assessed in triplicates. The standard error was evaluated for each experimental group. Blast2GO software (http://www.blast2go.com/b2ghome) was used to annotate all identified proteins using Gene Ontology (GO terms) and molecular function, biological process, and cellular component were determined. Mapping of DEPs and pathway analysis were carried out through Kyoto Encyclopedia of Genes and Genomes (KEGG) pathway database (http://www.kegg.jp/kegg/pathway.html)^74^. The protein-protein interaction network obtained using input into Cytoscape version 2.8.3.

## Electronic supplementary material

Supplementary Information - Table S5, Figure S1 and File S1

Supplementary information - Table S1-S4
