# Supplementary material for: Proteomic approach to understand the molecular physiology of symbiotic interaction between Piriformospora indica and Brassica napus
Source: Sci Rep. 2018 Apr 10;8:5773. doi: 10.1038/s41598-018-23994-z (PMC5893561; doi:10.1038/s41598-018-23994-z)
Supplement: Supplementary file 1 — Supplementary Information - Table S5, Figure S1 and File S1 [file 41598_2018_23994_MOESM1_ESM.pdf]

Proteomic approach to understand the molecular physiology of symbiotic interaction between *Piriformospora indica* and *Brassica napus*

Neeraj Shrivastava[1,7], JiangLi[2], Pan Li [2], Archana Kumari Sharma[3], Xingyuan Luo[4], Sanling Wu[5], Rashmi Pandey[6], Qikang Gao[2,5], Binggan Lou[1*]

[1]Institute of Biotechnology, Zhejiang University, Hangzhou 310058, China.

[2]Institute of Insect Sciences, Zhejiang University, Hangzhou 310058, China

[3]Centre for Environmental Science and Technology, Central University of Punjab, Bathinda 151001, India

[4]Hangzhou No. 14 High School, Hangzhou 310006, China

[5]Analysis Center of Agrobiology and Environmental Sciences, Zhejiang University, Hangzhou 310058, China

[6]Government Kanya Ashram School, Aara, Jashpur, Chhattisgarh, 496331, India

[7]Amity Institute of Microbial Technology, Amity University Uttar Pradesh Noida UP, 201303, India

*** Correspondence to bglou@zju.edu.cn**

SUPPLEMENTARY INFORMATION

Supplementary Table S1 Total proteins identified during the plant-fungal interaction.

Supplementary Table S2 Significant differentially expressed proteins (DEPs) in control vs *P.indica* treated samples.

Supplemental Table S3 Gene Ontology (GO) enrichment analyses of *P. indica* inhabited *B. napus* host's differential proteins.

Supplementary Table S4 List of DEPs proteins involved in Protein-protein interaction (PPI) network

(Supplementary Table 1-4 is provided separately in MS excel format)

Supplementary Table S5 List of DEPs showing role in different metabolic and synthesis pathways

Supplementary Figure S1 Tentative model of pre-symbiotic signaling cascade during recruitment of *P. indica* at rapeseed root tissue

Supplementary file S1: Summary - result statistics of protein identification.

S.N	KEGG map no.	Pathways	Differentially expressed Proteins	UniProt Accession
1.	00052	Glucose Metabolism	Acid beta-fructofuranosidase (3.2.1.26)	MAF6E3
2.	00061	Fatty Acid Metabolism	3-oxoacyl-[acyl-carrier-protein] (FabB) (2.3.1.41)	A0A0D3E4R7
			3-hydroxyacyl-[acyl-carrier-protein] dehydratase FabZ-like (4.2.1.59)	M4CPB4
3.	00220	Arginine Biosynthesis	N-acetyl-gamma-glutamyl-phosphate reductase (1.2.1.38)	A0A078ILF0
			Aminoacylase 1 (3.5.1.14)	A0A0D3CNP2
4.	00230	Purine Metabolism	Adenylosuccinate synthase (6.3.4.4)	A0A078EYR1
5.	00250	Alanine, Aspartate and Glutamate Metabolism	Adenylosuccinate synthase (6.3.4.4)	A0A078EYR1
6.	00500	Starch and Sucrose Metabolism	Acid beta-fructofuranosidase (3.1.2.26)	MAF6E3
7.	00511	Other Glycan Degradation	Alpha Mannosidase (2 manα1)	A0A078CNZ6
8.	00564	Glycerophospholipid Metabolism	Glycerophosphodiester phosphodiesterase (3.1.4.46)	M4ECA0
9.	00780	Biotin Metabolism	3-oxoacyl-[acyl-carrier-protein] (FabB) (2.3.1.41)	A0A0D3E4R7
10.	00940	Phenylpropanoid Biosynthesis	Peroxidase	C7E9R5

Supplementary Table S5. List of DEPs showing role in different metabolic and synthesis pathways

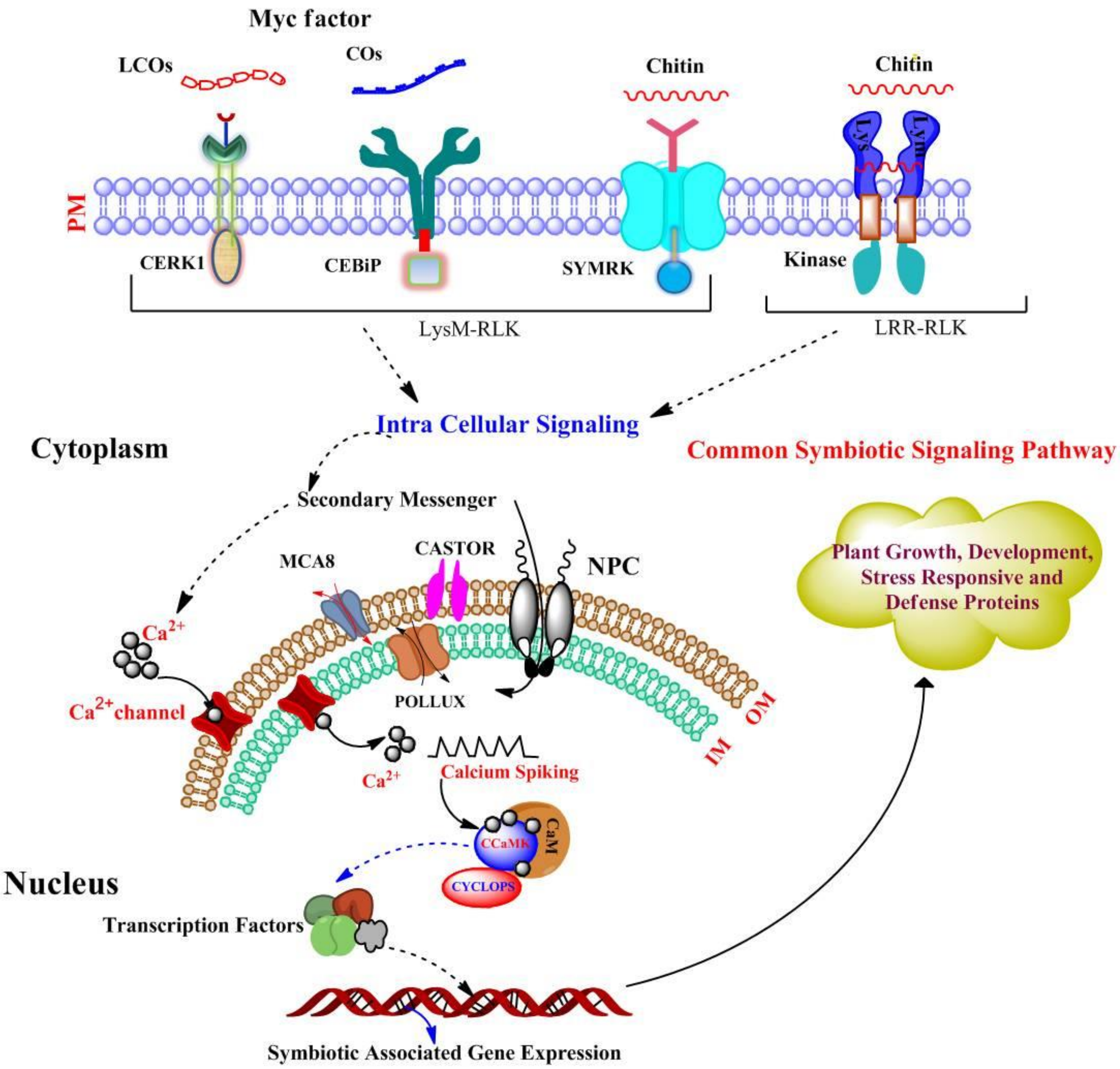

Supplementary Figure S1 Tentative model of pre-symbiotic signaling cascade during recruitment of *P. indica* at rapeseed root tissue: Microbe-associated molecular patterns (MAPS) on plasma membrane are responsible for recruiting different cytoplasmic regulatory receptor kinases. Germinated spores secrete chitin tetramers, CO4/Myc factors/LCOs. These ligands perception is mediated by LRR- receptor kinases (SYMRK), LysM receptors or receptor like proteins which trigger the recruitment of related SERK and CERK1.The LCO perception at the plasma membrane generate signal for nuclear Ca^{2+}-spiking vibrations. SYMRK and others generate second messenger and help in releasing calcium from the nuclear membranes. These events lead to initiate the nuclear calcium spiking response in nuclear membrane and mediated by upstream SYMRK/DMI2, CASTOR/POLLUX/DMI1) or downstream (CCaMK/DMI3, CYCLOPS/IPD3 etc.) transcription complexes. These factors induce the gene expression, which lead to arbuscule development which is major determinant of quantitative nutrient transfer. Further, expression of genes lead to form proteins engaged in overall growth and development of plant[28,29,30,31].

Supplementary file S1: Summary - Result statistics

(exported from PEAKS studio 8.0)

A) Protein identification

Table 1. Statistics of data.

Number of MS scans	24300
Number of MS/MS scans	271975

Table 2. Result filtration parameters.

Peptide -10lgP	≥20
Protein -10lgP	≥20
Proteins unique peptides	≥0
De novo ALC Score	≥50%

Table 3. Statistics of filtered result.

Peptide-Spectrum Matches	82303
Peptide sequences	18357
Protein groups	4410
Proteins	8123
Proteins (#Unique Peptides)	980 (>2); 814 (=2); 2525 (=1);
FDR (Peptide-Spectrum Matches)	0.8%
FDR (Peptide Sequences)	3.2%
FDR (Protein)	5.5%
De Novo Only Spectra	109,003

B) Label-free quantification

Figure 1: Protein profile heatmap (Cell colour represents the log2(ratio) to the average area across different samples)

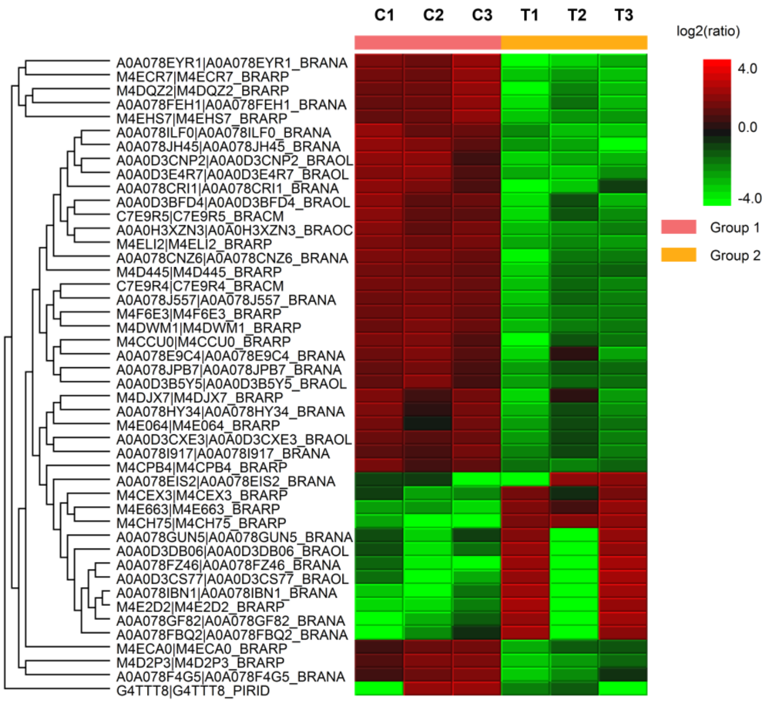

Figure 2. The volcano plot for proteins.

(a)

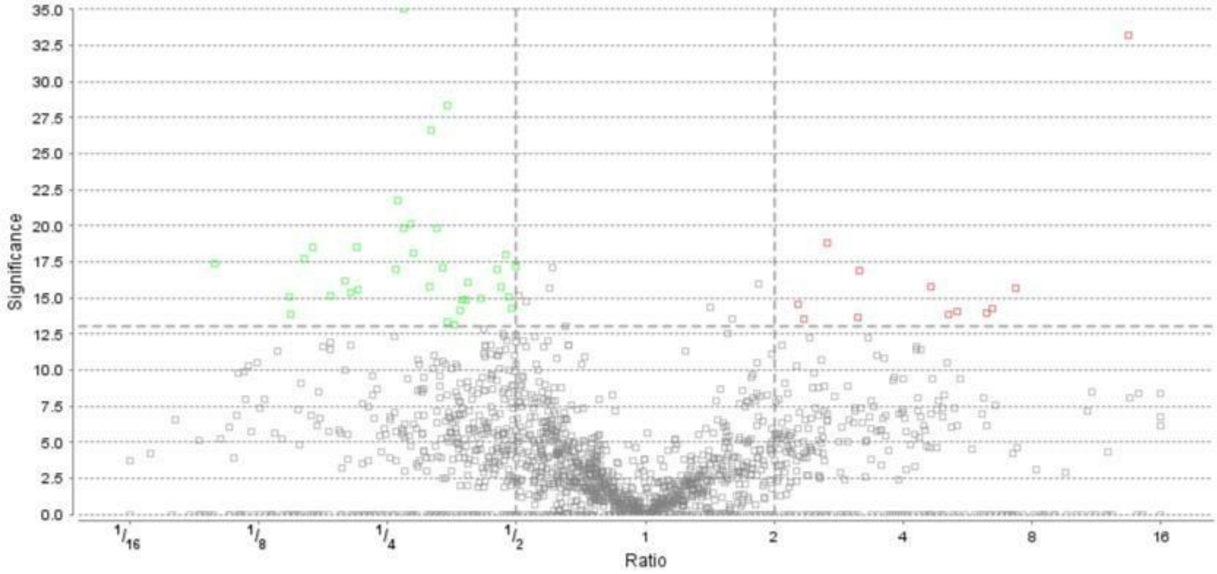

Figure 3. The distribution of feature vector ratio: (a) By quality. (b) By intensity.

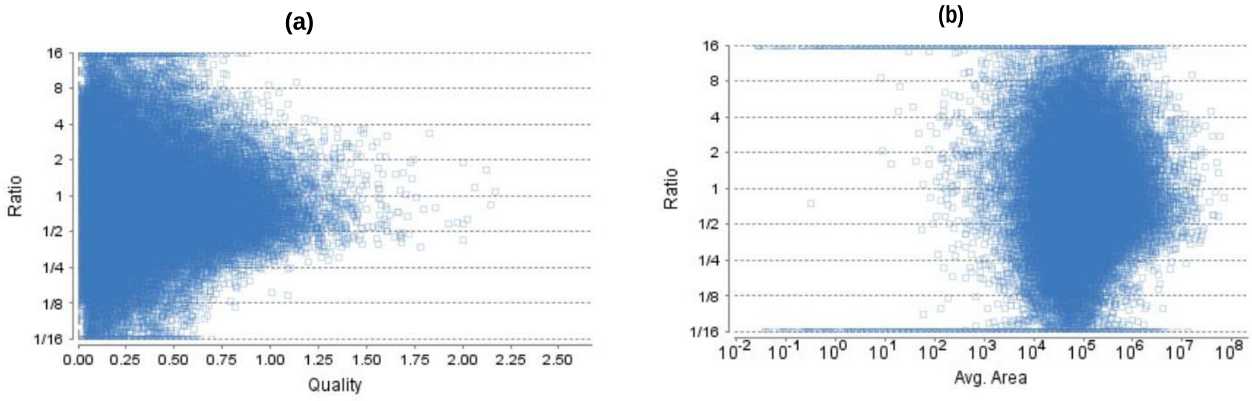

Figure 4. (a) RT shift distribution; (b) M/Z shift distribution.

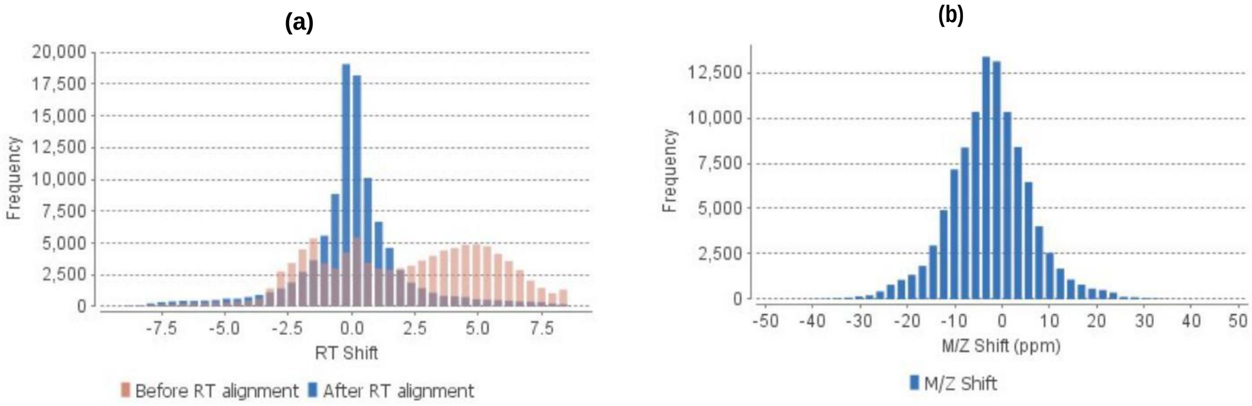

Table 1. Result filtration parameters.

Retention time	≥0
Retention time	≤150
Feature significance	≥0
Feature fold change	≥1
Quality	≥0
Avg. Area	≥0E0
Charge	≥1
Charge	≤10
Confident sample number	≥1
With peptide ID	true
Protein significance	≥13
Protein fold change	≥2
Significance method	ANOVA
Confident unique supports	≥1
Normalization	Use TIC

Table 2. Statistics of filtered result.

Features	44159
Features with ID	27217
Feature vectors	11364
Feature vectors with ID	11364
Protein groups	46

Table 3. Search Parameters

Quantification type: Label free quantification
Mass Error Tolerance: 25.0 ppm
Retention Time Shift Tolerance: 8.0 min
Dependent on PID: 19
FDR Threshold: 1%
Samples: 6 samples in 2 groups
: CK1 CK2 CK3 : T1 T2 T3
Reference Sample: CK2 (auto detected)
Training Samples: CK2, T2 (auto detected)
